# Targeting women with free cervical cancer screening: challenges and lessons learnt from Osun state, southwest Nigeria

**DOI:** 10.11604/pamj.2016.24.319.9300

**Published:** 2016-08-19

**Authors:** Ebenezer Gbenga Adepoju, Temitope Ilori, Samuel Anu Olowookere, Ajibola Idowu

**Affiliations:** 1 Department of Preventive Medicine, State Specialist Hospital, Osogbo, Osun state, Nigeria; 2 Department of Preventive Medicine and Primary Care, College of Medicine, University of Ibadan, Ibadan, Oyo state, Nigeria; 3 Department of Community Health, Faculty of Clinical Sciences, College of Health Sciences, Obafemi Awolowo University, Ile-Ife, Osun state, Nigeria; 4 Department of Community Medicine, Faculty of Clinical Sciences, Bowen University Teaching Hospital, Ogbomoso, Oyo State, Nigeria

**Keywords:** Free screening, cervical cancer, women, HPV vaccine, challenges, lesion learnt

## Abstract

**Introduction:**

The study was conducted to determine the challenges and suggest solutions to conducting free cervical cancer screening among Nigerian women.

**Methods:**

Awareness was created among women groups and mass media in Osun State for women to undergo free cervical cancer screening programme. Consenting women had their socio-demographic characteristics, awareness and uptake of HPV vaccine documented and papanicolaou smear procedure done with adequate referral for treatment given where necessary.

**Results:**

A total of 287 women had cervical cancer screening. Mean (SD) age was 51.6 (14.3) years. Most participants were urban based (87.1%), married (63.1%), had secondary education (39%) and were traders (79.1%). None of the women were aware of the preventive HPV vaccine or had been vaccinated against HPV. About 6% were pre-invasive while 0.7% had invasive cervical cancer. The highest proportions of respondents affected were young, married and had lower education. Challenges identified included poor attendance, low risk perception and logistic issues.

**Conclusion:**

Most participants were urban based. There is need to decentralize cancer of cervix screening through mobile clinics and establishment of screening centres in the rural areas. Neighbour to neighbour sensitization is essential. Also, HPV vaccine should be available and affordable to all girls before sexual maturity.

## Introduction

Cervical cancer is a malignant neoplasm of the cervix uteri which is a female reproductive organ that forms the lower portion of the uterus or womb [1–3] The cervix forms the part of the birth canal that opens to the vagina [1, 2]. It is a major public health problem throughout the world [3]. It is the commonest gynaecological cancer and the second leading cause of cancer death after breast cancer worldwide [3]. The World Health organization (WHO) estimates that cancer of cervix is responsible for up to 35% of adult female death [4]. Cervical cancer remains the commonest malignancy in Nigeria. About 370-500,000 cases are newly diagnosed annually with most cases occurring in developing countries [3]. In Nigeria, most cervical cancer cases is diagnosed in the advanced stages where only palliative care can be given to the affected patient [3]. Various studies on cervical cancer screening uptake among university students, market women and female health workers reported that though awareness about this deadly disease was high, uptake of screening had been abysmally low [5–7]. The management of the Osun State Ministry of Health with the support of the State government organized free cervical cancer screening for all women at the State Specialist Hospital, Osogbo. This study documents findings among recipients who were screened during the program.

## Methods

Osun state located in Southwest Nigeria; consist predominantly of the Yoruba ethnic group. Its population by 2006 census was 3,416,959 with women constituting half of this estimate. This prospective study of women resident in the state that attended the cervical cancer screening program sponsored by the State government and other non governmental organisations with the aim of increasing awareness about the deadly cancer and ensuring its early screening and detection. Awareness was created among women through various women groups and the State mass media about the free cervical cancer screening. These women were attended to by physicians, gynecologists and other health workers after their written consent was taken. Each participant's sociodemographic characteristics, clinical and gynecological history, awareness and uptake of HPV vaccine were documented. They were examined and had papanicolaou smear with colposcopy. Clients with positive papanicolaou smear or suspected cervical cancer were referred for definitive management. Information obtained was entered into SPSS version 16. Data analyzed with frequency for categorical variables, mean and standard deviation for continuous variables. Chi-square statistics was used to measure associations with p value <0.05 accepted as significant. Ethical consideration included taking written consent from participants and taking permission from the State Ministry of Health. Confidentiality was maintained with data collected kept in a password protected computer. Ethical approval was obtained from the State Ministry of Health Ethics and Research Committee.

## Results

A total of 287 women were screened. Mean (SD) age was 51.6 (14.3) years with majority (48.1%) in the 45-64 age group. Most participants were urban based (87.1%), married (63.1%), and had secondary education (39%). The majority of the participants were traders (79.1%) and farmers (19.9 %). Most were in monogamous relationship (71.1%) (Table 1). None of the women were aware of the preventive HPV vaccine or had been vaccinated against HPV. Only 3% have had previous cervical cancer screening. Table 2 reported the prevalence of cervical cancer among participants. Seventeen (6%) were diagnosed with pre-invasive cervical cancer while 2 (0.7%) participants had invasive cervical cancer. Table 3 reported the relationship between socio demographic variables and cervical cancer screening result among participants. The highest proportions of respondents affected were young, had lower education and were married. Challenges identified included poor attendance, low risk perception and logistic issues.

**Table 1:** Sociodemographic characteristics of participants

Characteristics	Frequency (N=287)	%
**Age group (years)**		
21-44	84	29.3
45-64	138	48.1
65 and above	65	22.6
**Marital status**		
Single	18	6.3
Married	181	63.1
Divorced	21	7.3
Widow	67	23.3
**Level of education**		
None	65	22.6
Primary	53	18.5
Secondary	112	39.0
Tertiary	57	19.9
**Religion**		
Christianity	133	46.3
Islam	154	53.7
**Occupation**		
Unemployed	3	1.0
Trading	227	79.1
Farming	57	19.9
**Place of abode**		
Urban	250	87.1
Rural	37	12.9

**Table 2:** Result of cervical screening among participants

Variable	Frequency (N=287)	%
Pre-invasive	17	6.0
Invasive	2	0.7
Normal	268	93.3

**Table 3:** Relationship between sociodemographic variables and cervical screening result among participants

Variable	Papanicolaou smear result		p-value^+^
	Positive(%) n=19	Negative(%) n=268	
**Age group (years)**			
21-44	8 (9.5)	76 (90.5)	0.203
≥45	11 (5.4)	192 (94.6)	
**Education**			
None/primary	10 (8.5)	108 (91.5)	0.291
Secondary/tertiary	9 (5.3)	160 (94.7)	
**Marital status**			
Married	14 (7.7)	167 (92.3)	0.321
Single/divorced/widow	5 (4.7)	101 (95.3)	

+ Pearson’s chi-square

## Discussion

This study reported the outcome of a free cervical cancer screening among Nigerian women. It showed that uptake of cervical cancer screening was low since most women did not come for the program despite the public sensitization. Various studies had reported such attitude among women [8–10]. Possible reasons for staying away could be ineffective awareness creation, socioeconomic as well as not realizing the importance of this program.

Most women that attended were urban based. Public transport could have been provided at strategic places especially for women resident in rural areas to convey these women from their place of abode to the hospital. Moreover the screening could be decentralized and community based to encourage these women to attend. Also mobile screening could be done. In addition, incentive such as food could be provided to encourage attendance. Also some women may not be aware of the program hence there might be need to do more neighbour to neighbour sensitization for such program in future. Also, it is necessary to target religious leaders, other opinion leaders, their spouses and other decision makers in future programs.

This study showed that all age group above 18 years were affected. This agreed with findings in previous studies [3, 5, 6]. This had been shown to be due to exposure to prevalent risk factors to developing cervical cancer. Also, none of the women were aware of the preventive HPV vaccine. This showed that awareness should be created in females before onset of sexual intercourse to take the HPV vaccine to prevent development of cervical cancer. Studies had proven that some strains of HPV causes cervical cancer and that the vaccine protects if given to young girls before sexual debut [2, 10]. Emphasis should be placed on availability and affordability of this vaccine to all girls before sexual maturity. About 6% of the participants had pre-invasive cervical cancer while 0.7% had invasive cervical cancer. Although this is low compared with previous studies [1, 2]. It nevertheless confirmed that cervical cancer is a health problem among the study population that must be curbed.

A limitation of this study is that the study population is a convenience sample of women residents who attended the cervical cancer screening program hence it will be difficult to generalize its findings to all women in the state. Hence a state wide screening program is encouraged to determine the true prevalence of this deadly condition. A cervical cancer registry should be established in the state to document the burden of the disease.

## Conclusion

In conclusion, cervical cancer is a problem that must be curbed. Most participants that attended were urban based hence need to decentralize cervical cancer screening through mobile clinics and establishment of screening centres in the rural areas. Neighbour to neighbour sensitization on cervical cancer screening is essential. Also, HPV vaccine should be available and affordable to all girls before sexual maturity.

### What is known about this topic

- Cervical cancer is a common killer of women;
- Routine screening ensure early diagnosis and treatment.

### What this study adds

- Cervical cancer was reported among the participants;
- It is essential to extend affordable cervical cancer screening to all women through integrating routine and mobile screening at all community health care centres; Also there is need for neighbour to neighbour sensitization on need to prevent cervical cancer through routine papanicolaou smear at all health centres in the state and targeted community screening;
- HPV vaccine should be available and affordable to all girls before sexual maturity.

## References

[1] Ferlay J, Bray F, Pisani P, Parkin DM (2004). GLOBOCAN 2002: Cancer incidence, mortality and prevalence worldwide. IARC Cancer Base No. 5, Version 2.0.

[2] De Vuyst H, Lillo F, Broutet N, Smith JS (2008). Review HIV, human papillomavirus, and cervical neoplasia and cancer in the era of highly active antiretroviral therapy. Eur J Cancer Prev..

[3] Ahdieh L, Muñoz A, Vlahov D, Trimble CL, Timpson LA, Shah K (2000). Cervical neoplasia and repeated positivity of human papillomavirus infection in human immunodeficiency virus-seropositive and -seronegative women. Am J Epidemiol..

[4] Konopnicki D, Manigart Y, Gilles C, Barlow P, de Marchin J, Feoli F, Larsimont D, Delforge M, De Wit S, Clumeck N (2013). High-risk human papillomavirus infection in HIV-positive African women living in Europe. J Int AIDS Soc..

[5] Couture MC, Page K, Stein ES, Sansothy N, Sichan K, Kaldor J, Evans JL, Maher L, Palefsky J (2012). Cervical human papillomavirus infection among young women engaged in sex work in Phnom Penh, Cambodia: prevalence, genotypes, risk factors and association with HIV infection. BMC Infect Dis..

[6] Santos C, Muñoz N, Klug S, Almonte M, Guerrero I, Alvarez M, Velarde C, Galdos O, Castillo M, Walboomers J, Meijer C, Caceres E (2001). HPV types and cofactors causing cervical cancer in Peru. British Journal of Cancer.

[7] Olowookere SA, Abioye-Kuteyi EA, Airewele EP, Fasure HA, Fayose O, Onakpoma F, Ibitoye A (2012). Awareness and uptake of HPV vaccination and cervical cancer screening among female undergraduate students in a Tertiary Institution in Nigeria. Nigerian Journal of Family Practice.

[8] Adekanle DA, Adeyemi AS, Afolabi AF (2011). Knowledge, Attitude and Cervical Cancer Screening Among Female Secondary School Teachers in Osogbo, Southwest Nigeria. Academic Journal of Cancer Research.

[9] Ayinde OA, Omigbodun AO (2003). Knowledge, attitude and practices related to prevention of cancer of the cervix among female health workers in Ibadan. J Obstet Gynaecol..

[10] Ogunbode OO, Ayinde OA (2005). Awareness of cervical cancer and screening in a Nigerian female market population. Annals of African Medicine.

